# Cryopreservation of shoot apices and callus cultures of globe artichoke using vitrification method

**DOI:** 10.1186/s43141-019-0016-1

**Published:** 2020-01-13

**Authors:** Shawky A. Bekheet, Valbona Sota, Hattem M. El-Shabrawi, Alaa M. El-Minisy

**Affiliations:** 1 Plant Biotechnology Department, National Research Center, Cairo, Egypt; 2 Department of Biotechnology, Faculty of Natural Sciences, University of Tirana, Tirana, Albania

**Keywords:** Cryopreservation, Globe artichoke, Tissue culture, Vitrification

## Abstract

**Background:**

Cryogenic cooling became a crucial tool for the storage of *heterozygous plants such as* globe artichoke. This study was carried out to optimize a reliable method for in vitro cryopreservation of shoot apices and callus cultures of globe artichoke using dimethylsulfoxide (DMSO) and Plant Vitrification Solutions 2 (PVS2) as cryoprotectant solutions. Shoot apices were exposed to DMSO or PVS2 for 20, 40, 60, and 80 min prior to plunge in liquid nitrogen (LN).

**Results:**

It was found that using PVS2 as a cryoprotectant in cryopreservation of shoot apices of globe artichoke was more effective compared with using of DMSO alone. Among the exposure time tested, 60 min gave the best results of survival. The highest survival (60%), regeneration (56%), and proliferated shootlets (4.30) were obtained after cryoprotection with PVS2 for 60 min. Regarding callus cultures, the maximum values of fresh weights and subsequently growth value of recovered callus were registered with 40 min followed by 60 min exposure time. Related to the type of the tested cryoprotectants, the best survival and growth parameters of the cryopreserved callus cultures were obtained with PVS2 treatments. Treatment with PVS2 for 40 min registered the highest survival observations of cryopreserved callus. Also, the maximum values of fresh weight (1.30 g) and growth value (4.20) were obtained with 40 min exposure time. Microscopy analysis presented as cell morphology revealed that the treatment of PVS2 40% was the optimum for cell growth of cryopreserved callus of globe artichoke.

**Conclusion:**

The results demonstrated that using PVS2 as a cryoprotectant in cryopreservation of shoot apices and callus cultures of globe artichoke was more effective compared with DMSO.

## Background

Globe artichoke (*Cynara scolymus* L.) plant, the native to the Mediterranean area is cultivated for its nutritional and medicinal values. This species has a complex sexual reproduction. When they have been propagated by seed, the yield of their progeny is frequently different from that of the parental generation. Vegetative propagation using suckers and axillary buds is commonly used for multiplication of globe artichoke plant materials. However, the low rate of multiplication and the potential for disseminating diseases are two major factors that hinder the expansion and development of globe artichoke through vegetative propagation. Biodiversity of globe artichoke represents a valuable genetic resource that needs suitable propagation and conservation methods. At the present, artichoke germplasm is preserved in field as active and base collections which attacked by pests and pathogens, and entail high costs of labor and technical staff [1]. The high variability of seeds, together with difficulties in the distribution and exchange of healthy plant material from field, support tissue culture and cryopreservation as being the best alternative approaches for germplasm conservation of globe artichoke. In vitro culture techniques have been used not only for clonal propagation and but also used for germplasm conservation [2]. Medium-term preservation is achieved by reducing the growth of plant material, thus increasing intervals between subcultures. For long-term conservation, cryopreservation (liquid nitrogen, − 196°C) allows storing plant material without modification or alteration for extended periods and protected from contaminations. At this temperature, all cellular divisions and metabolic processes are stopped. It is worth to mention that cryopreservation of shoot tips is favoring for long-term and high genetic stability germplasm conservation. Otherwise, in vitro preservation of plant cells or callus cultures is a valuable tool not only for the conservation of genetic resources, but also to preserve plant materials in a form ready for further manipulations such as in vitro production of secondary metabolites [3]. Cryogenic cooling is considered an effective option for long term-storage of undifferentiated plant cells as a source of phytochemicals and for other manipulations.

Cryopreservation methods are different and include the older classic methods based on freeze-induced dehydration of cells as well as newer methods based on vitrification. Vitrification involves treatment of plant samples with cryoprotective substances, dehydration with highly concentrated solutions, rapid cooling and rewarming, removal of cryoprotectants, and recovery. This procedure has been developed for shoot apices and somatic of many plant species [4, 5]. However, classical techniques have been applied to undifferentiated cultures, such as cell suspension cultures and calli. In vitrification technique, the cryoprotectant molecules provide the optimum cellular cryoprotection environment of plant cells. In this respect, dimethylsulfoxide (DMSO) is the most common chemical pretreatment. It is used both for pregrowth and during cryoprotection [6, 7]. DMSO reduces the electrolytic concentration in the residual chilled contents in and around of a biological cell, during cryopreservation. Practically, PVS2 (Plant Vitrification Solution n° 2) consists of 30% glycerol, 15% ethylene glycol, 15% dimethylsulfoxide (DMSO) (all v/v), and 0.4 M sucrose is the most applied solution [8]. This solution is more effective for dehydration and less toxic to plant material [9]. For successful vitrification procedures, it is essential to carefully control the cryoprotectant to provide enough dehydration while at the same time preventing injury caused by chemical toxicity and sudden osmotic stress [10]. This study aimed to develop a simple and efficient cryopreservation method for long-term storage of shoot apices and callus cultures of globe artichoke using DMSO and Plant Vitrification Solutions 2 (PVS2) as cryoprotectants.

## Methods

### Establishment in vitro cultures

**Fig. 1:**
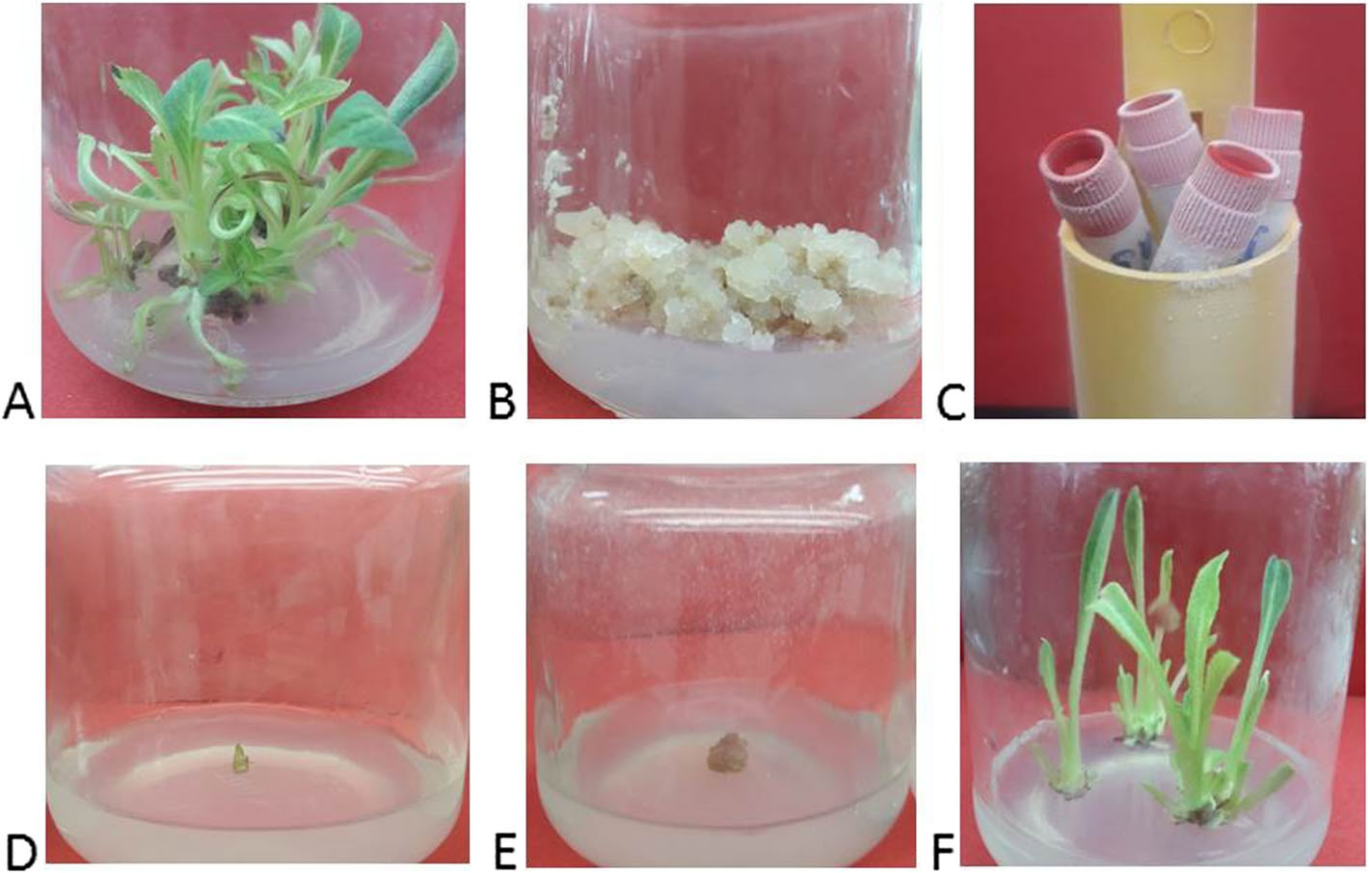
Shootlets of globe artichoke grown on MS medium supplemented with 1 mg/l BA (**a**), callus cultures were initiated from leaf explants and maintained on MS medium amended with 0.1 mg/l NAA, 0.5 mg/l 2,4-D, and 0.5 mg/l kin (**b**). Cryotubes taken from liquid nitrogen container (**c**), cryopreserved shoot apices grown on MS medium + 1 mg/l BA (**d**), cryopreserved callus inoculum transferred onto MS medium + 0.1 mg/l NAA, 0.5 mg/l 2,4-D, and 0.5 mg/l kin (**e**) and shootlets developed from cryopreserved shoot apices after 12 weeks of culturing on the recovery medium (**f**)

Shootlets cultures of globe artichoke (Green Globe cultivar obtained from Vegetable Research Department, Horticulture Research Institute, Agricultural Research Center, Egypt) were in vitro established on Murashige and Skoog [11] (MS) medium supplemented with 1 mg/l 6-benzyladenine (BA) according to method developed by Bekheet et al. [12]. Subcultures were performed every 4–5 weeks, in order to get enough mother stock cultures (Fig. 1a). Callus cultures were initiated from leaf explants as described by Bekheet et al. [13] and maintained on MS medium amended with 0.1 mg/l Naphthaleneacetic acid (NAA), 0.5 mg/l 2,4-Dichlorophenoxyacetic acid (2,4-D), and 0.5mg/l Kinetin (kin) by transferring onto fresh medium four weeks intervals [Fig. 1b].

### **Cryopreservation procedure**

Shoot apices (1 cm) and callus inocula (250 mg) of globe artichoke taken from in vitro grown cultures (after the third subculture) were used as explants for cryopreservation experiments. The explants were precultured on filter-sterilized loading solution (2 M glycerol and 0.4 M sucrose dissolved in MS medium) using Petri dishes and incubated at 25 °C for 24 h. Shoot apices were directly immersed into the solutions, while callus inocula were wrapped in a small strip of sterilized cotton. After the loading treatment, the explants were transferred into 2 ml polypropylene sterile cryovials and exposed to 10% of dimethylsulfoxide (DMSO) in MS medium or Plant Vitrification Solutions 2 [PVS2; 15*% (*w/v) ethylene glycol, 15% (w/v) DMSO, 30% (w/v) glycerol, 0.4 M sucrose in MS medium, Sakai et al. [8] for different exposure times, i.e., 20, 40, 60, or 80 min at 0 °C. For each treatment at least ten cryotubes were employed. The explants were then suspended into cryotubes with 0.5 ml fresh of the two vitrification solutions and the tubes were directly immersed into liquid nitrogen (LN) (at − 196 °C) and kept for at least 48 h.

### Thawing and recovery

After cryostorage, cryotubes were taken from liquid nitrogen container (Fig. 1c) and rapidly thawed in a beaker filled with sterile water at 40 °C for about 1.5 to 2 min until most of the ice has melted. After rewarming, the cryoprotection solution was removed from the cryotubes; the explants were rinsed with MS liquid medium for 20 min at room temperature and blotted on filter paper. Then, the explants were transferred onto recovery media [MS medium + 1 mg/l BA for shootlets regeneration (Fig. 1d) and MS medium + 0.1 mg/l NAA, 0.5 mg/l 2,4-D, and 0.5 mg/l kin for callus growth (Fig. 1e)], and then all the cultures were incubated under standard conditions of illumination and temperature.

### Survival and regrowth of cryopreserved shoot apices

Assessment of survival percentages and regeneration of the cryopreserved shoot apices were carried out after four weeks of culturing on the recovery medium. The survival was recorded (after 2 weeks) based on visual observation as the formation of green tissue developing from the shoot apices (%, no of green shoot apices/total no of shoot apices in freezing × 100). Regeneration was recorded (after 4 weeks of culturing on recovery medium) when a minimum of two leaves arise from the emerging bud ((%, no of shoot apices that elongated or produced new shoots/total no of shoots × 100). Also, a number of proliferated shootlets was recorded after culturing for 12 weeks on the regeneration medium.

### Survival and regrowth of cryopreserved callus cultures

Four weeks after inoculation on callus maintenance medium, survival was evaluated based on visual observation by examining the colors of callus cultures. The creamy ones (with increase of the volume) had survived; the brown ones had died. Also samples for each treatment were subjected to regrowth. Fresh weight and growth value of the survival callus cultures were determined after 8 weeks of inoculation.

$$ \mathrm{Growth}\ \mathrm{Value}=\frac{\mathrm{Final}\ \mathrm{fresh}\ \mathrm{weight}-\mathrm{Initial}\ \mathrm{fresh}\ \mathrm{weight}}{\mathrm{Initial}\ \mathrm{fresh}\ \mathrm{weight}} $$

### Microscopy

To assess the damage caused by the pre-treatment and freezing process, sample of the cryopreserved callus exposed for different times (20, 40, 60, or 80 min) to PVS2 were taken and then fixed in Karnovsky’s solution [14] at room temperature before being washed in 0·05 m Sörensen phosphate buffer pH 72. Samples were progressively dehydrated through ethanol solutions to a final concentration of 100 % and then photographed under a light microscope (Nicon, Japan Labophot-2 Microscope) equipped with KL 2500 LCD light sources (Nicon, Japan) was used to analyze the cell growth.

### Tissue culture medium and incubation conditions

Tissue culture media were solidified with 0.7% agar and supplemented with 30 g/l sucrose, 100 mg/l myo-inositol, 1 mg/l pyridoxine-HCl, 1 mg/l nicotinic acid and 0.2 mg/l thiamine-HCl. The pH was adjusted to 5.8 before autoclaving at 121 °C and 1.5 Ib/M^2^ for 25 min. In all treatments, the growth regulators were added to the culture medium prior to autoclaving. Cultures were normally maintained at 25 ± 2 °C and 16 h photoperiod provided by white fluorescent tubes (3000 lux light intensity).

### Experimental design and statistical analysis

The experiments were set up as a separate completely randomized design and repeated two times using. Data were statistically analyzed using standard error (SE) according to the method described by Snedecor and Cochran [15]. The results are presented as means of 25 replicates.

## Results

### Cryopreservation of shoot apices

#### Effect of exposure to dimethylsulfoxide on recovery

Suitable cryoprotectants and explants are very effective in enhancing survival of the cryopreserved plant material. In this experiment shoot apices (the most commonly plant material used for direct shoot formation) was used as explants for cryopreservation of globe artichoke. To determine the optimal time of exposure to dimethylsulfoxide (DMSO) on recovery, shoot apices were treated with DMSO for 20, 04, 60, and 80 min prior to a plunge in LN. Data obtained revealed that exposure to DMSO for various durations resulted in a variable rate of survival and regeneration percentages as well as number of proliferated shootlets (Table 1). Surviving shoot apices turned green, while dead ones became white or brown after two weeks of post-thaw culture. The highest survival (36%) and regeneration (32%) percentages were obtained from shoot apices treated with DMSO for 60 min. However, treatment with DMSO for 40 min gave maximum number of proliferated shootlets (3.50) after 12 weeks of culturing on recovery medium. On contrast, exposure to DMSO for 80 min gave lowest recovery parameters presented as survival, regeneration, and number of proliferated shootlets. With this treatment, surface of the most cryopreserved shoot apices turned to dark brown color and did not form new shoots or green leaf. The results proved that prolonged exposure (80 min) to the DMSO solution appeared to be harmful for explants, while 20-min treatment was not enough to protect the explants during ultra-rapid freezing (Table 1).

**Table 1:** Effect of exposure time to dimethylsulfoxide on recovery of cryopreserved shoot apices of globe artichoke.

Exposure time (min)	Survival (%)	Regeneration (%)	Number of proliferated shootlets
20	20	16	2.70 ± 0.05
40	24	20	3.50 ± 0.12
60	36	32	3.00 ± 0.10
80	16	12	2.50 ± 0.15

±standard error (SE)

#### Effect of exposure to PVS2 on recovery

This experiment was carried out to evaluate the effect of exposure time to PVS2 on survival and regeneration rates, as well as shootlets proliferation of cryopreserved shoot apices of globe artichoke. The recovery potential of cryopreserved shoot apices was investigated on MS media supplemented with 1 mg/l BA. Our results indicated that different exposure periods to PVS2 had a strong influence on the growth parameters after cryostorage. In general, all the growth parameters gradually increased as exposure time to PVS2 increased till 60 min and then decreased (Table 2). The highest survival (60%), regeneration (56%), and proliferated shootlets (4.30) were obtained after cryoprotection with PVS2 for 60 min before immersing into liquid nitrogen. With 40 min exposure treatment, it was noticed that all the survived shoot apices (40%) regenerated into shootlets without necrosis. However, when explants were exposed to PVS2 for 80 min only, most of the shoot apices turned brown and then died and only 24% survived. After cryopreservation, the explants grew very slowly during the first 2 weeks. Shoot apices resumed growth and proliferation within next 10 weeks, and no morphological abnormalities were observed in the developed shootlets (Fig. 1f). So, it could be concluded that the best response was obtained for shoot apices treated for 60 min in PVS2 resulting in highest survival with well-developed shootlets.

**Table 2:** Effect of exposure time to PVS2 on recovery of cryopreserved shoot apices of globe artichoke

Exposure time (min)	Survival (%)	Regeneration (%)	Number of proliferated shootlets
20	32	24	3.00 ± 0.10
40	40	40	4.00 ± 0.17
60	60	56	4.30 ± 0.12
80	24	20	3.50 ± 0.20

±standard error (SE)

### Cryopreservation of callus cultures

#### Effect of exposure to dimethylsulfoxide on regrowth

In this experiment, callus cultures of globe artichoke were subjected to different periods of exposure to dimethylsulfoxide (DMSO) (10%) as cryoprotectant before plunging into LN. The post cryopreservation growth capacity of callus was evaluated. The results reveal that regrowth patterns of cryopreserved callus differed from the exposure period to DMSO. The highest fresh weight (1.00 g) and growth value (3.00) were obtained with 40 min exposure to DMSO. It was found that there was a trend towards a dramatic reduction in callus survival with increasing exposure periods more than 40 min (Table 3). Increasing exposure duration above 40 min also led to decreasing both fresh weight and growth value. No survival at all was observed with 80 min exposure to DMSO. It seems this period causes high desiccation or toxicity for the cells. Otherwise, significant differences in terms of fresh weight of cryopreserved calli were obtained with the 20 and 40 min periods. This illustrates the importance of exposure period length to DMSO as a cryoprotectant solution. Based on this experiment, it appears that exposure to 40 min is considered to be the best DMSO treatment for cryopreservation of globe artichoke callus by vitrification. These findings can be explained by the fact that undifferentiated cells need shorter treatment with DMSO to be sufficiently dehydrated.

**Table 3.** Regrowth of cryopreserved callus of globe artichoke exposed for different time to dimethylsulfoxide

Exposure time (min)	Survival	Fresh weight (g)	Growth value
20	+	0.80 ± 0.11	2.20
40	++	1.00 ± 0.15	3.00
60	+	0.60 ± 0.20	1.40
80	−	0.25 ± 0.01	0.00

±standard error (SE), − no survival, + low, ++ medium frequency of survival

#### Effect of exposure to PVS2 on regrowth

This experiment was conducted to optimized exposure time of globe artichoke callus cultures to PVS2 in order to have successful survival upon cryopreservation. The explants were treated with PVS2 for 20, 40, 60, or 80 min at 0 °C as a step of cryopreservation. Data indicated that survivability of cryostored callus was obviously affected by the time of exposure to PVS2. Also, the amount of callus growth in term of callus fresh weight was significantly influenced by exposure periods. Exposure to PVS2 for 40 min gave the highest survival observations. Also, the maximum values of fresh weight (1.30 g) and subsequently growth value (4.20) were registered also with 40 min followed by 60 min exposure time Table 4. The differences between fresh weights obtained from the two treatments were statistically significant. However, the lowest growth parameters were obtained when callus was exposed to PVS2 for 80 min before plunge into LN. It was noticed that white new cells was formed from the callus after a 4-week culture on recovery medium (MS medium + 0.1 mg/l NAA, 0.5 mg/l 2,4-D and 0.5 mg/l kin) while unviable calli turned brown. The maximum growth of callus was obtained after 8 weeks of culturing on the recovery medium. The recovered callus cultures were friable and ranging in color from white to brown. The results proved that the time exposure to PVS2 is critical for survivability of globe artichoke callus cryopreserved by vetrification. Comparing to DMSO, using of PVS2 as cryoprotectant was found to be very efficient for the cryopreservation of globe artichoke callus cultures.

**Table 4:** Regrowth of cryopreserved callus of globe artichoke exposed for different time to PVS2.

Exposure time (min)	Survival	Fresh weight (g)	Growth value
20	++	1.00 ± 0.11	3.00
40	+++	1.30 ± 0.10	4.20
60	++	1.10 ± 0.15	3.40
80	+	0.70 ± 0.12	1.80

±standard error (SE), − + low, ++ medium, and +++ high-frequency survival

**Fig. 2:**
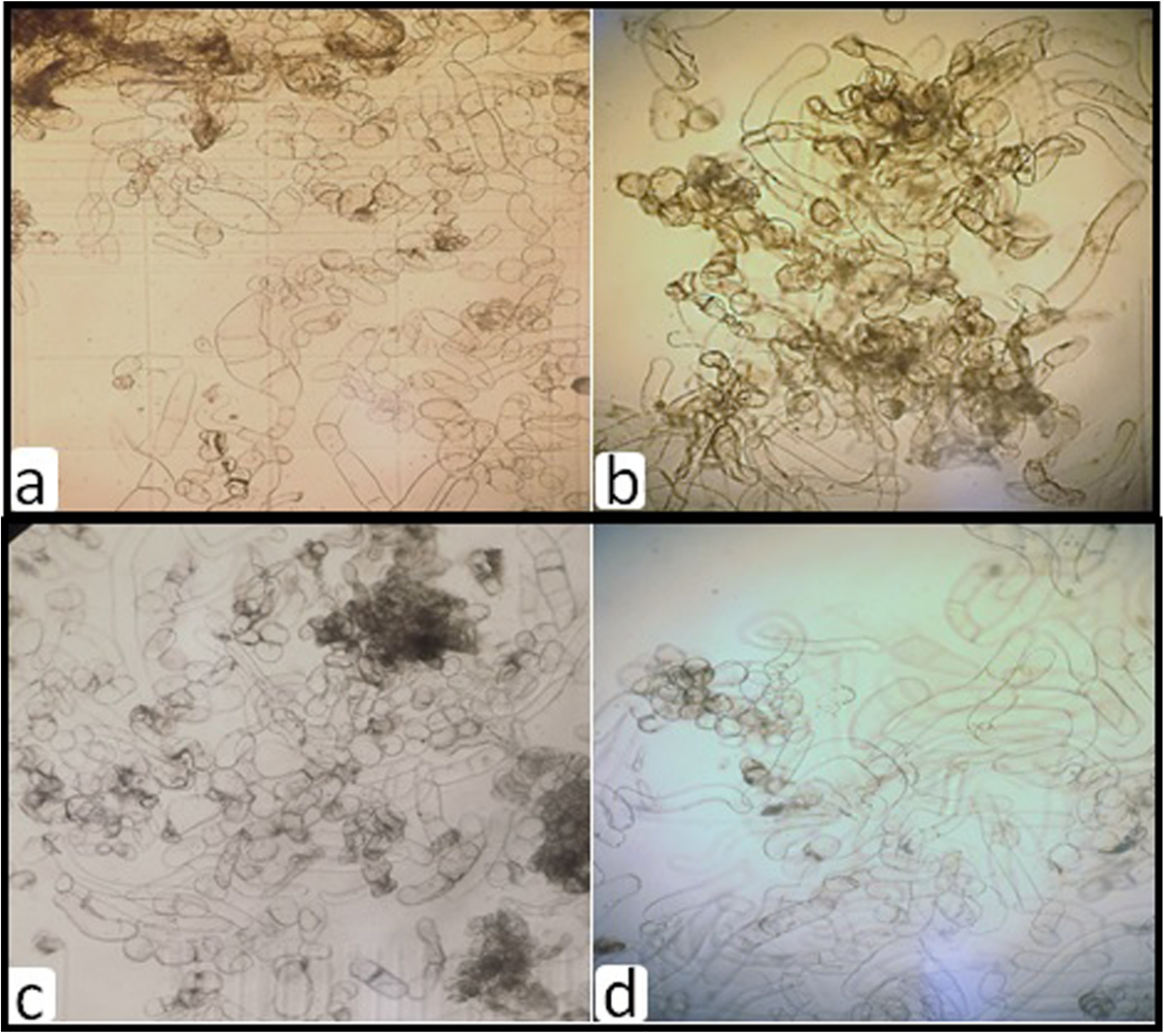
Morphological characterization of globe artichoke cells after cryopreservation treatments. **a** 20, **b** 40, **c** 60, **d** 80 min exposure time to PVS2 using light microscope

On the other hand, the microscope data analysis confirmed the growth results (Fig. 2). The cell morphology revealed that the treatment of exposure to PVS2 for 40 min was the optimum for cell growth. Almost all of the cells were in perfect spherical shape with several cell clusters which indicate to healthy growth shape and appear in good condition comparing to the lowest PVS2 time (20 min) (Fig. 2-b). However, exposure to 80 min of PVS2 was not good at all for cell growth after recovery since most of the cells character morphology was very showing elongated cell shapes and forming no clustering phenotype (Fig. 2-d).

## Discussion

The vitrification protocol can be used for the wide range of species with complex tissue structure such as shoot tips and embryos which contain a variety of cell types [16]. In this technique, tolerance to cryoprotectant solutions is acquired by optimizing the pre-conditioning and loading treatments, as well as the duration of exposure to the solution. In this respect, DMSO alone, at a concentration of 5–10%, is often reported as a cryoprotectant [17–19]. Other authors prefer combinations of cryoprotectants at lower concentrations, considering this approach more beneficial than a single cryoprotectant at high concentration. Usually, dimethyl sulfoxide is the main penetrating colligative cryoprotectant and it may be combined with non-penetrating osmotically active additives such as sugars, polyethylene glycol, and polyvinylpyrrolidone [7]. The most widely vitrification procedure is the treatment with PVS2 solution for periods ranging from 30 to 90 min [6]. Due to possible toxic effects of the PVS2 solution which can compromise plant cell viability, the exposure time is a fundamental parameter which must always be optimized. In this context, shoot tips, which consist of small and dense cells with low water content, are often chosen as basic plant material for cryopreservation, but they are also widely used due to their high genetic stability**.**

In the present work, time of exposure to DMSO or PVS2 showed statistical differences in recovery (survival and regeneration percentages as well as number of proliferated shootlets) of cryopreserved shoot apices of globe artichoke. Among all exposure time tested, 60 min resulted in the highest recovery parameters. The results also, demonstrated that using of PVS2 as cryoprotectant in cryopreservation of shoot apices of globe artichoke was more effective comparing with using of DMSO alone. The positive found effect of PVS2 may be due to higher dehydration obtained by the combination of four compounds, glycerol, ethylene glycol, DMSO, and sucrose. The reduction in regeneration compared to survival percentage may be attributed to partial damage of the cells due to osmotic shock or dehydration. It is important to mention that there was no survival following cryopreservation without treatment with cryoprotectants either DMSO alone or PVS2 solution (in primary experiments). It is apparent from the results that, in addition to the type of cryoprotectant, the treatment time also played an important role in survival. The present results are accordance with those obtained by Tavazza et al. [1]. In their study on cryopreservation (based on vitrification) of globe artichoke shoot tips of two spring cultivars “Grato 1” and “Campagnano” they reported that the best survival percentage of cryopreserved shoot tips was 61% for “Grato1” and 55% for “Campagnano.” They added, 30 min exposure time is not effective in protecting the tips during cooling. Recently, Zhang et al. [20] optimized a method for cryopreservation of four Jerusalem artichoke cultivars using shoot tip explants. However, Sakai and Engelmann [4] reported that time duration of exposure to PVS2 was found critical for success of cryopreservation by vitrification-based cryogenic protocols and optimal time duration varies from cryogenic procedures and plant species. In this respect, Niino et al. [21] mentioned that the survival rate of the vitrified shoot tips of cherry cultivar Sendaiya increased gradually with the duration of exposure to PVS2 and reached the maximum value after 90 and 105 min of exposure. However, optimal time durations of exposure to PVS2 for shoot regrowth of cryopreserved buds of potato were 5–7 h [22].

In order to obtain successful survival callus cultures upon cryopreservation by vitrification, chemical compositions of the cryoprotectant and its exposure time should be optimized to dehydrate the cytoplasm without damaging plant cells. A mixture of cryoprotectants vitrifies easier upon direct immersion in liquid nitrogen compared to solutions with only one cryoprotectant in a higher concentration [23]. In the present study, we measured the post cryopreservation growth presented as fresh weight and growth value to evaluate the morphogenetic capacity of cryopreserved callus cultures of globe artichoke. Our findings show that, with cryopreserved callus regrowth changed with respect to exposure duration to DMSO or PVS2. Exposure for 40 min to DMSO or PVS2 gave the best survival. Further, fresh weight and growth value took same trend. It was found that, exposure to PVS2 registered best survival and growth parameters of the cryopreserved callus cultures of globe artichoke comparing with DMSO. Accordingly, PVS2 appears to be more suitable than DMSO for the cryopreservation of globe artichoke callus. The positive role of PVS2 was also demonstrated on successful regeneration of cryopreserved *Thymus moroderi* Pau ex Martı´nez after exposure for 60 min [24] and 30 min [25]. In contrast, Al-Bahrany and Al-Khayri [26] mentioned that DMSO is more suitable for the cryopreservation of date palm cv. Khalas as assessed by the amount of callus re-growth. In another species, Mathur et al. [27] reported that survival of cryopreserved embryogenic cultures of *Pinus roxburghii* was best achieved using 0.3 M sorbitol combined with 5% DMSO. In this respect, it has been demonstrated that embryogenic callus and cell cultures survived after being subjected to a number of cryoconservation treatments including conventional cryoprotection with dimethylsulfoxide (DMSO) followed by slow cooling [28], vitrification followed by fast cooling [8, 9], as well as other simplified procedures [29, 30]. On the other hand, cryoprotectant solution has been found to have characteristic temperature dependent exposure time. It is 3 min at 25 °C for cultured cells of navel orange [29], 7.5 min at 0 °C for rice embryogenic cells [31], and 15 min at 0 °C for meristems of white clove [32]. The morphological data presented as cell shape confirm our quantitative result of the growth value. Such cell morphological and structural parameters were used to determine cell damage of cryopreserved plant tissues [33].

## Conclusion

Our findings revealed that PVS2 solution was more effective for cryoprotection of in vitro grown tissue cultures of globe artichoke. Treatment with PVS2 for 60 min resulting in highest survival and well-developed shootlets of cryopreserved shoot apices. However, 40 min exposure to PVS2 was enough for successful cryopreservation of callus cultures.

## Data Availability

The data are available from Plant Biotechnology, Department, National Research Center, Egypt, and material is not applicable.

## Abbreviations

2,4-D: *2,4-Dichlorophenoxyacetic acid*
BA: 6-Benzyladenine
DMSO: Dimethylsulfoxide
NAA: Naphthaleneacetic acid
PVS2: Plant Vitrification Solutions 2
